# A Manual of Operative Surgery

**Published:** 1878-08

**Authors:** 


A Manual of Operative Surgery. By Lewis A. Stinson, B.A. (Yale), M.D., Surgeon to the Presbyterian Hospital, Professor of Pathological Anatomy in the Medical Faculty of the University of New York, with three hundred and thirty-two illustrations. Philadelphia: Henry C. Lea. 1878.

This work consists of one volume of 476 pages, and seems admirably adapted to the requirements of the practical surgeon. The various operations are succinctly described and plainly illustrated; and while the book does not take the place of text-books on the science and art of surgery, it will be found more useful as a hand-book in practice.

